# Supplementary material for: Investigation on Antibiotic-Resistance, Biofilm Formation and Virulence Factors in Multi Drug Resistant and Non Multi Drug Resistant Staphylococcus pseudintermedius
Source: Microorganisms. 2019 Dec 16;7(12):702. doi: 10.3390/microorganisms7120702 (PMC6956092; doi:10.3390/microorganisms7120702)
Supplement: Supplementary file 1 [file microorganisms-07-00702-s001.pdf]

International Journal of
Molecular Sciences

MDPI

Supplementary Table 1. Phenotypic and genetic determination of biofilm-forming ability.

non MDR strains			MDR strains		
ID	PCR *icaA*/*icaD*	MtP	ID	PCR *icaA*/*icaD*	MtP
3	+/+	Weak	4	+/+	Moderate
6A	+/+	Weak	12	+/+	Strong
7B1	+/+	Moderate	14	+/+	Strong
8	+/+	Moderate	27	+/+	Weak
10A	+/+	Weak	28A	+/+	Strong
11B	+/+	Weak	28B	+/+	Strong
13	+/+	Weak	238	+/+	Moderate
16	+/+	Moderate	34	+/+	Moderate
18A	+/+	Weak	35	+/+	Strong
19A	+/+	Moderate	36	+/+	Strong
20	+/+	Weak	37	+/+	Strong
23	+/+	Weak	39	+/+	Strong
26A	+/+	Absent	41	+/+	Strong
30B	+/+	Moderate	42	+/+	Strong
31	-/-	Moderate	45	+/+	Strong
48	+/+	Weak	46	-/+	Strong
51	+/+	Weak	54	+/+	Strong
53	+/+	Weak	56	+/+	Strong
55	+/+	Weak	58	+/+	Moderate
59	+/+	Weak	60	+/+	Moderate
61	+/+	Weak	62	+/+	Strong
70	+/+	Absent	63	-/+	Moderate
74	+/+	Weak	64	-/+	Strong
79	+/+	Moderate	66	-/+	Moderate
84	+/+	Moderate	67	+/+	Moderate
85	+/+	Weak	71	+/+	Moderate
91	+/+	Moderate	72	-/+	Strong
96	+/+	Absent	76	+/+	Moderate
98	+/+	Strong	77	-/+	Moderate
234	+/+	Weak	78	-/+	Moderate
502	+/+	Absent	81	-/+	Moderate
			82	+/+	Moderate
			87	-/+	Moderate
			89	-/+	Moderate
			93	+/+	Weak
			94	-/-	Strong
			99	-/+	Strong
			102	-/+	Moderate
			104	-/+	Moderate
			105	+/+	Moderate
			107	+/+	Moderate
			117	+/+	Strong
